# Similar Supine Heart Rate Variability Changes During 24-h Exposure to Normobaric vs. Hypobaric Hypoxia

**DOI:** 10.3389/fnins.2021.777800

**Published:** 2021-12-09

**Authors:** Valérian Tanner, Raphael Faiss, Jonas Saugy, Nicolas Bourdillon, Laurent Schmitt, Grégoire P. Millet

**Affiliations:** ^1^Medicine School, Faculty of Biology and Medicine, University of Lausanne, Lausanne, Switzerland; ^2^Institute of Sport Sciences, University of Lausanne, Lausanne, Switzerland; ^3^REDs, Research and Expertise in Anti-Doping Sciences, University of Lausanne, Lausanne, Switzerland; ^4^National Centre of Nordic-Ski, Research and Performance, Prémanon, France

**Keywords:** heart rate variability, arterial oxygen saturation, normobaric normoxia, hypobaric hypoxia, normobaric hypoxia, autonomic nervous system, altitude

## Abstract

**Purpose:** This study aimed to investigate the differences between normobaric (NH) and hypobaric hypoxia (HH) on supine heart rate variability (HRV) during a 24-h exposure. We hypothesized a greater decrease in parasympathetic-related parameters in HH than in NH.

**Methods:** A pooling of original data from forty-one healthy lowland trained men was analyzed. They were exposed to altitude either in NH (F_I_O_2_ = 15.7 ± 2.0%; PB = 698 ± 25 mmHg) or HH (F_I_O_2_ = 20.9%; PB = 534 ± 42 mmHg) in a randomized order. Pulse oximeter oxygen saturation (S_p_O_2_), heart rate (HR), and supine HRV were measured during a 7-min rest period three times: before (in normobaric normoxia, NN), after 12 (H12), and 24 h (H24) of either NH or HH exposure. HRV parameters were analyzed for time- and frequency-domains.

**Results:** S_p_O_2_ was lower in both hypoxic conditions than in NN and was higher in NH than HH at H24. Subjects showed similarly higher HR during both hypoxic conditions than in NN. No difference in HRV parameters was found between NH and HH at any time. The natural logarithm of root mean square of the successive differences (LnRMSSD) and the high frequency spectral power (HF), which reflect parasympathetic activity, decreased similarly in NH and HH when compared to NN.

**Conclusion:** Despite S_p_O_2_ differences, changes in supine HRV parameters during 24-h exposure were similar between NH and HH conditions indicating a similar decrease in parasympathetic activity. Therefore, HRV can be analyzed similarly in NH and HH conditions.

## Introduction

Altitude training is an effective method commonly used by elite athletes (Millet and Brocherie, 2020). Prolonged altitude exposure increases erythropoiesis, which is explained by increased plasma erythropoietin concentration (Stray-Gundersen et al., 2001) and leads to an apparent increase in peripheral tissues oxygen delivery. This altitude-related increase in red blood cell mass increases the oxygen-carrying capacity of the blood and improves aerobic power (Hauser et al., 2016). Moreover, skeletal muscle adaptations could improve oxygen extraction during altitude exposition (Brooks et al., 1992).

The two main methods used for hypoxic exposure are hypobaric hypoxia (HH; FIO2 = 20.9%; PB <760 mmHg; real altitude in mountain or hypobaric “decompression” chamber) and normobaric hypoxia (NH; FIO2 <20.9%; PB = 760 mmHg; simulated altitude by using hypoxic facilities). On the one hand, HH has been used for years by endurance athletes during training camps On the other hand, the democratization of NH, accelerated by technical progress, allows the use of the usual lowland infrastructures, and avoids the time and inconvenience of transport to altitude. An updated panorama of all HH and NH hypoxic training methods is available in Girard et al. (2017). Based on the equivalent air altitude equivalent model (Conkin and Wessel, 2008), NH is frequently used as a surrogate to HH. However, recent research suggested that NH and HH are not interchangeable (Fulco et al., 2011; Millet et al., 2012; Saugy et al., 2014, 2016a; Coppel et al., 2015; DiPasquale et al., 2015a; Conkin, 2016) although the differences between HH and NH are still debated (Millet and Debevec, 2020; Richalet, 2020). The following differences were already reported for a matched inspired oxygen pressure (P_I_O_2_) in HH vs. NH, respectively: (1) lower pulse oximeter oxygen saturation (S_p_O_2_) (Saugy et al., 2016c; Aebi et al., 2020; Debevec et al., 2020); (2) greater performance impairment (Beidleman et al., 2014; Saugy et al., 2016a), both recently confirmed in a study with perfectly controlled environmental factors (Takezawa et al., 2021); (3) higher oxidative stress (Faiss et al., 2013; Ribon et al., 2016); (4) impaired nitric oxide bioavailability (Faiss et al., 2013); (5) greater fluid retention (Loeppky et al., 2005; Conkin and Wessel, 2008), and (6) sleep structure perturbation (Heinzer et al., 2016; Saugy et al., 2016b). In addition, breathing patterns are affected as well: lower tidal volume, lower minute ventilation, higher physiological dead space, and higher respiratory frequency during HH exposure compared to NH (Savourey et al., 2003; Conkin and Wessel, 2008; Saugy et al., 2014). It was also demonstrated that HH provokes stronger hypoxic pulmonary vasoconstriction, altering the ventilation-perfusion ratio (Loeppky et al., 1997). Moreover, hypocapnia and blood alkalosis are greater in HH than NH (Savourey et al., 2003; Coppel et al., 2015). Despite all these differences, which suggest that exposure in terrestrial altitude (HH) may induce more pronounced stress with larger physiological responses than simulated altitudes (NH), there is still an ongoing debate about their clinical significance. A “point-counterpoint” (Millet et al., 2012; Mounier and Brugniaux, 2012) reported the main arguments on this subject, and a more recent crosstalk (Millet and Debevec, 2020; Richalet, 2020) brought the debate back to the forefront. All protagonists agreed that further studies with a rigorous methodological approach and new perspectives would allow a more comprehensive understanding of the role of barometric pressure *per se* in different hypoxic conditions.

One of the potential perspectives to evaluate the differences between HH and NH could be the heart rate variability (HRV). HRV is the fluctuation in the time intervals between adjacent heartbeats (Malik et al., 1996). There are two main methods for the HRV analyses: time-domain and frequency-domain. Time-domain indices of HRV (LnRMSSD, natural logarithm of root mean square of the successive differences) quantify the amount of variability in measurements of the interbeat interval. Frequency-domain measurements [The low frequency spectral power (LF) and HF, respectively low and high frequency spectral power] estimate the distribution of absolute or relative power into frequency bands. This non-invasive method is used to assess cardiac autonomic control (Buchheit, 2014). It is especially an accurate means of estimating parasympathetic activation by looking specifically at HF with frequency-domain analysis (Malik et al., 1996; Grossman and Taylor, 2007). Moreover, those frequency-domain analyses are known to be more sensitive than time-domain (Schmitt et al., 2015a). In general, acute hypoxia decreases HRV and parasympathetic activity (Wille et al., 2012). Since S_p_O_2_ is lower in HH, the hypoxic chemoreflex should lead to a greater vagal withdrawal in HH than in NH (Parati et al., 2015) and thus potentially result in differences in the autonomic cardiac control. Moreover, variations in S_p_O_2_ are related to variations in HRV parameters. Indeed, a decrease in S_p_O_2_ is related to a decrease in both LnRMSSD (Krejèí et al., 2018) and HF and to an increase in the low-to-high frequency ratio (LF/HF) (Botek et al., 2015). Since ventilation influences cardiac autonomic activity (Brown et al., 1993), the above-mentioned differences in breathing patterns between NH and HH could also generate distinct HRV responses. Considering those physiological mechanisms, one may speculate on different influences of NH vs. HH on HRV.

The effects of NH vs. HH on HRV were studied only during acute exposures (≤ 30 min) (Basualto-Alarcón et al., 2012; Aebi et al., 2020). The LF/HF ratio was slightly higher in HH, but the evidence for a hypobaric effect *per se* on HRV in hypoxia during acute exposures was not strong enough (Aebi et al., 2020). Therefore, the present study aimed to compare the influence of a 24-h exposure in NH vs. HH on supine HRV. Since HH induced a larger desaturation than NH, the hypothesis of a greater decrease in parasympathetic-related HRV parameters (such as LnRMSSD and HF) in HH condition was tested. Furthermore, since breathing patterns are different between HH and NH, greater differences in frequency-domain parameters (especially in HF influenced by respiratory sinus arrhythmia) (Grossman and Taylor, 2007) than in time-domain parameters (i.e., LnRMSSD) were expected.

## Methods

### Subjects

Forty-one healthy lowland trained men regularly practicing endurance sports have participated in our study. The main characteristics of all subjects were: age 30 ± 7 years, body height 179 ± 5 cm, body weight 73 ± 7 kg, VO_2_max 62 ± 8 mL⋅kg^–1^⋅min^–1^. Subjects were included only if exposed to the three NN, NH, and HH conditions. They were non-smokers and neither acclimatized nor recently exposed to altitude for at least a month before the experiment. Subjects’ characteristics are displayed in Table 1.

**TABLE 1:** Data are reported as mean ± standard deviations.

	Faiss et al., 2013	Saugy et al., 2016a	Saugy et al., 2016c
Subjects	12	13	16
Age (years)	35 ± 8	34 ± 9	24 ± 4
Bodyweight (kg)	74 ± 8	76 ± 7	70 ± 5
Height (cm)	179 ± 7	180 ± 4	179 ± 5
VO_2_max (mL⋅kg^–1^⋅min^–1^)	61 ± 7	60 ± 10	66 ± 8
**Protocol**			
Total exposure time (h)	24	26	432
Washout period (days)	23	12	347
Measuring time points used (h)	H0, H8, H12, H20, and H24	H0, H10, and H20	H0, H24
**NN**			
Altitude (m)	485	485	1150
Barometric pressure (mmHg)	720 ± 1	718 ± 4	665 ± 11
F_I_O_2_ (%)	20.9	20.9	20.9
P_I_O_2_ (mmHg)	140.0 ± 1.2	140.5 ± 0.6	140.1 ± 1.0
Temperature (°C)	24 ± 1	23 ± 1	22 ± 2
**NH**			
“Simulated” altitude (m)	3000	3450	2250
Barometric pressure (mmHg)	720.0 ± 1.0	715.8 ± 3.8	666.6 ± 3.6
F_I_O_2_ (%)	14.7 ± 0.1	13.6 ± 0.0	18.1 ± 0.0
P_I_O_2_ (mmHg)	99.0 ± 0.4	91.0 ± 0.6	111.9 ± 0.6
Temperature (°C)	27 ± 2	23 ± 1	22 ± 3
**HH**			
Altitude (m)	3000	3450	2250
Barometric pressure (mmHg)	530.0 ± 6.0	481.8 ± 4.7	580.2 ± 2.9
F_I_O_2_ (%)	20.9	20.9	20.9
P_I_O_2_ (mmHg)	102.0 ± 0.3	90.9 ± 1.0	111.6 ± 0.6
Temperature (°C)	25 ± 2	21 ± 1	20 ± 4

*VO2max, maximal oxygen uptake; Washout period, minimum period between the two hypoxic exposures; NN, normobaric normoxia, control condition few hours before each hypoxic exposure; NH, normobaric hypoxia; HH, hypobaric hypoxia; FIO2, fraction of inspired oxygen; PIO2, partial pressure of inspired oxygen.*

### Study Design

This study brings together original unanalyzed data from three previous studies performed by our laboratory (Faiss et al., 2013; Saugy et al., 2016a,c). All these studies have in common a randomized continuous 24 h exposure in NH and HH for each subject and a comparison with the control condition (NN). The measurements in NN were performed a few hours before each hypoxic exposure, whether in NH or HH. Regarding the washout period that was different between the two hypoxic exposures (see Table 1), the minimum duration required has always been respected to avoid potential interactions between hypoxic exposures. Many more methodological details are reported in the already published studies, but their respective main characteristics are also outlined in Table 1 for the reader’s convenience.

Since these studies were planned independently and their measurement times do not match exactly, measurement time points were grouped into the following three categories:

• **NN:** H0 (all three studies, control condition before each hypoxic exposition).
• **H12:** H8 and H12 (Faiss et al., 2013); H10 (Saugy et al., 2016a).
• **H24:** H24 (Saugy et al., 2016c); H20 and H24 (Faiss et al., 2013); H20 (Saugy et al., 2016a).

### Measurements

Measurements included S_p_O_2_, heart rate (HR), and supine HRV with identical equipment and measurement protocol between studies. S_p_O_2_ and HR were recorded continuously using a wrist oximeter connected to a finger sensor (Wristox 3100TM with 8000SM-WO Sensor; Nonin, Plymouth, MN) at each measurement time point during a 7 min supine rest period at 0.25 Hz.

The HRV measurement was performed during the last 5 min of a 7 min supine rest period. Measurement of the interval duration between two R waves of the cardiac electrical activity was performed with an HR monitor. Heartbeats that do not originate from the sino-atrial node have been shown to have drastic effects on the outcome of HRV indexes (Malik et al., 1996). To this end, the RR-intervals were first analyzed to remove ectopic beats from the recordings using automatic and visual inspections of the RR series. Then, time-domain analyses were performed with LnRMSSD, reflecting mainly the activity of the parasympathetic system (Kleiger et al., 2005; Plews et al., 2013). The spectral power was calculated with Fast Fourier Transform and was expressed in ms^2^: with HF (0.15–0.40 Hz) reflecting modulation of parasympathetic influence to the heart (Malik et al., 1996; Shaffer et al., 2014) and related to respiratory sinus arrhythmia (Grossman and Taylor, 2007); and LF (0.04–0.15 Hz) reflecting mainly baroreceptor activity during resting conditions (Goldstein et al., 2011; Shaffer et al., 2014; Mccraty and Shaffer, 2015). LF was calculated in absolute spectral power units (ms^2^) as described above and in normalized units (nu) with LFnu = LF/(LF + HF). This study does not show HFnu because of its total dependence with LFnu, both in absolute values and statistical analysis. The total spectral power (TP) was calculated by adding LF and HF. The LF/HF ratio was also calculated. All procedures were carried out according to the Task Force recommendations (Malik et al., 1996).

### Statistical Analysis

Data are reported as mean ± standard deviation in the text and table, mean ± standard error in graphs. Data were tested for both homogeneity of variance (Fisher-Snedecor *F*-test) and normality (Shapiro–Wilk test). When both conditions were met, a two-way measures analysis of variance (ANOVA) was performed to assess whether physiological variables were differentially affected depending on the condition (NH, HH) and time points (H0, H12, and H24). Tukey’s *post hoc* tests were used to localize differences when significant main or interaction effects were found. When either equality of variance or normality were not satisfied, variables were analyzed for each condition using a Friedman test for repeated measures to determine time effects using pairwise multiple comparison procedures (Bonferroni test). Differences between overall exposure mean (H12 and H24 combined) in NH vs. HH were compared using paired *t*-test. Corrections for multiple comparisons were applied (Bonferroni). Null hypothesis was rejected at *p* < 0.05. The statistical analyses were performed with SPSS 27.0 software (IBM Corp., New York, NY, United States).

## Results

The statistical power (calculated with G*Power software, version 3.1.9.7, Heinrich-Heine-Universität Düsseldorf) was satisfying for the four “main” variables in descending order; SpO2 (0.95); LnRMSSD (0.85); HR (0.74); and HF (0.69).

### Oxygen Saturation

**FIGURE 1:**
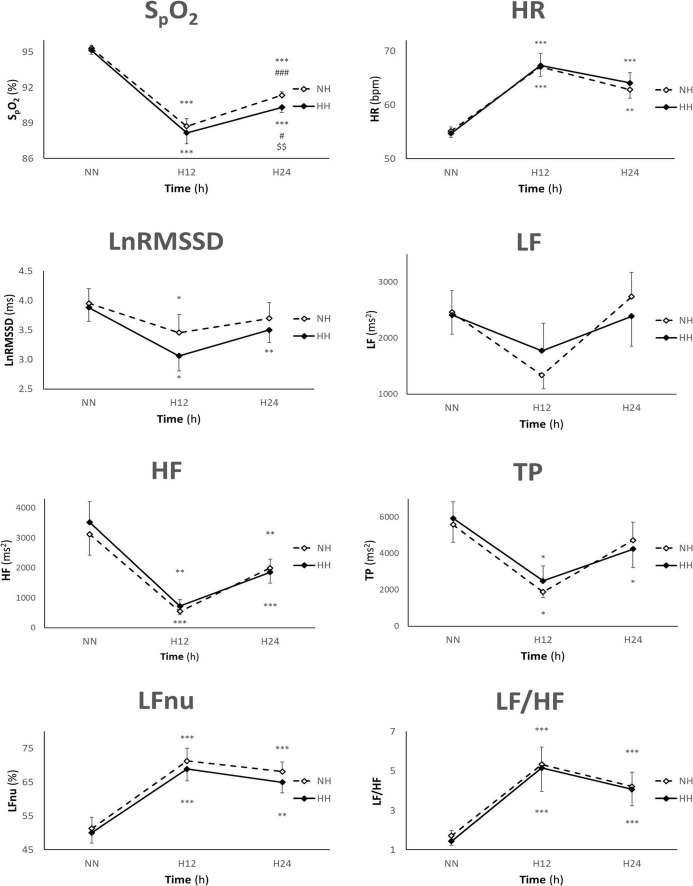
Values are presented as mean ± SE. *n* = 41, 25, and 41 for NN, H12, and H24, respectively (*n* = 36, 18, and 37 for S_p_O_2_). HR, heart rate; LnRMSSD, natural logarithm of the root mean square of the successive differences; LF, low frequency; HF, high frequency; TP, total power; NN, normobaric normoxia; NH, normobaric hypoxia; HH, hypobaric hypoxia; bpm, beat per minute; h, hours; *p < 0.05, **p < 0.01, and ***p < 0.001 for difference with NN, #*p* < 0.05, ^###^*p* < 0.001 for difference with H12, ^$$^*p* < 0.01 for difference with NH.

There were significant main effects on **S_p_O_2_** between exposures (*p* = 0.009) and time points (*p* < 0.001). The interaction was significant (*p* = 0.038). As expected, S_p_O_2_ decreased in both hypoxic conditions at H12 (*p* < 0.001) and H24 (*p* < 0.001) when compared to NN. S_p_O_2_ was higher in NH than in HH only at H24 (*p* = 0.002). S_p_O_2_ was higher at H24 than at H12 in both NH (*p* < 0.001) and HH (*p* = 0.021) (Figure 1). The overall exposure mean (H12 and H24 combined) yielded a higher S_p_O_2_ in NH vs. HH (*p* = 0.008).

### Heart Rate

There was a significant main effect on **HR** between time points (*p* < 0.001). HR increased in both exposures at H12 (*p* < 0.001, *p* < 0.001) and H24 (*p* < 0.001, *p* < 0.001) when compared to NN (Figure 1). No differences between NH and HH were found either in individual time or overall exposure mean (H12 and H24 combined).

### Heart Rate Variability

There were significant main effects between time points on all HRV parameters except LF. **LnRMSSD** was lower in both NH and HH at H12 (*p* = 0.044, *p* = 0.022), but only in HH at H24 (*p* = 0.008), when compared to NN (Figure 1). **LF** did not show any significant main effect (Figure 1). **HF** decreased in NH and HH at H12 (*p* < 0.001, *p* = 0.004) and H24 (*p* = 0.008, *p* < 0.001) when compared to NN (Figure 1). **TP** decreased in both exposures at H12 (*p* = 0.041, *p* = 0.041), but only in HH at H24 (*p* = 0.012) when compared to NN (Figure 1). **LFnu** increased in NH and HH at H12 (*p* < 0.001, *p* < 0.001) and H24 (*p* < 0.001, *p* = 0.003) when compared to NN (Figure 1). **LF/HF** ratio increased in both exposures at H12 (*p* < 0.001, *p* < 0.001) and H24 (*p* < 0.001, *p* < 0.001) when compared to NN (Figure 1). No differences in HRV parameters between NH and HH were found either in individual time or overall exposure mean (H12 and H24 combined).

## Discussion

To our knowledge, the present study is the first to compare heart rate variability between normobaric normoxia, normobaric hypoxia, and hypobaric hypoxia during prolonged exposures. The main finding was that NH and HH elicited similar HRV changes during 24-h exposures despite a higher mean S_p_O_2_ in NH. Indeed, the decrease in parasympathetic-related parameters (LnRMSSD and HF) was similar between NH and HH, contrary to our hypothesis.

To properly interpret the results of this study, it is necessary to mention the main cardiorespiratory adaptations in acute hypoxia: (1) a decrease in S_p_O_2_ due to reduction in inspired oxygen pressure (P_i_O_2_) (Maufrais et al., 2017), even at low altitude (Goldberg et al., 2012) and despite the diverse hypoxic-related ventilatory and cardiovascular compensations; (2) an increase in resting heart rate and cardiac output (Duplain et al., 1999; Bärtsch and Gibbs, 2007; Naeije, 2010), due to increased sympathetic activity and vagal withdrawal (Koller et al., 1988, 19) and inversely proportional to the decrease in S_p_O_2_ to maintain unchanged oxygen delivery to the tissues (Naeije, 2010); and (3) a largely unchanged blood pressure (Wolfel et al., 1994; Parati et al., 2015). The baroreflex and chemoreflex are also of great importance when considering the effects of hypoxia on the autonomic nervous system. On the one hand, the peripheral chemoreceptors in the carotid body respond primarily to hypoxemia and increase sympathetic activity while decreasing parasympathetic activity (Parati et al., 2015). On the other hand, the baroreflex ensures strict blood pressure regulation with stretch-sensitive baroreceptors located in the carotid sinuses and aortic arch. An increased blood pressure activates the baroreceptors, leading to decreased sympathetic activity and greater parasympathetic activation (Pitzalis et al., 1998). Finally, the already well-documented higher S_p_O_2_ in NH vs. HH can be explained by faster and shallower respiration, which increases dead space ventilation in HH (Faiss et al., 2013). The initial triggers of this S_p_O_2_ difference between NH and HH are still unclear. However, since chemosensitivity seems not to be changed in altitude (Loeppky et al., 1996), modification in gases density and diffusivity are the main hypotheses (Savourey et al., 2003). Our study confirmed a higher oxygen saturation in NH vs. HH after 24 h of hypoxic exposure. This is already well described for shorter exposition times (Aebi et al., 2020; Debevec et al., 2020) and logically was also already reported in the three support experiments of the present study. However, the latter was still debated for exposures longer than 8 h (Coppel et al., 2015). As expected, HR was higher in hypoxic conditions compared to normoxia in response to the increased sympathetic activity and vagal withdrawal. Several studies have further found lower HR in NH vs. HH (Savourey et al., 2003; DiPasquale et al., 2015b; Heinzer et al., 2016; Ross et al., 2019; Aebi et al., 2020) but only during short exposures. In addition, studies examining longer exposures (>8 h) found no difference in HR (Faiss et al., 2013; Richard et al., 2014). Unsurprisingly, our results hence suggest similar HR between NH and HH.

A decrease in LnRMSSD (Liu et al., 2001; Long et al., 2006) and HF (Bernardi et al., 1998; Sevre et al., 2001) in hypoxia is already well described and demonstrates a clear hypoxic-related parasympathetic decrease. Firstly, since LnRMSSD mainly reflects the activity of the parasympathetic system (Kleiger et al., 2005; Plews et al., 2013) and is not very sensitive to respiratory variations (Hill et al., 2009; Buchheit, 2014), its decrease in both hypoxic conditions at H12 was not surprising. When compared to NN, a potentially larger parasympathetic tone reduction might explain that LnRMSSD remained lower in HH but not in NH at H24. However, the difference between NH and HH was not significant (*p* = 0.328). Secondly, since vagal activity is the major contributor to the HF component (Malik et al., 1996; Shaffer et al., 2014), a significant decrease in both hypoxic exposures at H12 and H24 was observed as expected. HF is called the “respiratory band” because it corresponds to the frequency of the respiratory sinus arrhythmia variations (Grossman and Taylor, 2007). Therefore, considering the differences in respiratory patterns between NH and HH mentioned in the introduction, one may expect differences in HF between NH and HH. However, these differences remained non-significant, maybe due to limited breathing differences or other confounding factors (as discussed below in “*Strengths and limitations”*). Thus, considering the LnRMSSD and HF data, it can be concluded that parasympathetic activity is similarly decreased in NH and HH compared to NN.

Hypoxia increases sympathetic activity (Duplain et al., 1999) mainly *via* peripheral chemoreceptor activation (Heistad and Abboud, 1980), as proved by increased plasma and urinary catecholamines (Mazzeo et al., 1991; Bogaard et al., 2002) and skeletal muscle sympathetic activity (Hansen and Sander, 2003). Therefore, the similar increase in HR between NH and HH may be seen as a consequence of similar sympathetic activity between those two hypoxic conditions. However, since there is no well-established marker of sympathetic activity in the HRV parameters measured in this study, it is not possible to make a stronger statement about the sympathetic activity similarities in NH vs. HH here.

LF reflects baroreceptor activity during resting conditions (Goldstein et al., 2011; Shaffer et al., 2014; Mccraty and Shaffer, 2015) which is primarily vagally mediated (Keyl et al., 2001). Therefore, it should not be used as a marker of sympathetic nervous system activity at rest (Eckberg, 1983; Kember et al., 2001; Billman, 2013; Shaffer et al., 2014). Since hypoxia increases sympathetic activity partly through altered baroreceptor function (Hainsworth et al., 2007), and as already reported (Schmitt et al., 2015b,^2018^; Aebi et al., 2020), a decrease in LF during hypoxic exposures could theoretically be observed. However, that was not the case in this study since no difference in LF between normoxia and hypoxia was found, thus confirming the challenging interpretation of LF. Two additional precautions should be kept in mind when considering the data presented here. On the one hand, the consequent decrease in HF can alone be the reason behind the significant increase of LFnu in NH and HH exposures, especially considering that both branches of the autonomic nervous system are frequently non-reciprocal (Billman, 2013). On the other hand, it is often assumed that LF/HF ratio reflects the sympathetic-parasympathetic balance (Taylor, 2006). However, it is frequently shifted due to reductions in LF power which do not reflect sympathetic nervous system activity at rest. The LF/HF ratio should thus be interpreted with caution (Shaffer et al., 2014). Moreover, both branches of the autonomic nervous system can be simultaneously active (Berntson and Cacioppo, 1999). Furthermore, the interactions between parasympathetic and sympathetic nervous systems are complex, non-linear, and frequently non-reciprocal (Billman, 2013). Therefore, as LF is not decreased, it can be stated that this increase in LF/HF ratio mainly displays the hypoxic sympathetic-parasympathetic misbalance due mainly to a parasympathetic alteration.

Altogether, despite the greater hypoxemia in HH, which could lead to a potential greater decrease in parasympathetic activity, no differences in HRV between NH and HH were found. When taken as a whole, the above results converge toward lowered parasympathetic activity during hypoxic exposures and confirmed previous findings (Marshall, 1998; Wille et al., 2012), again without differences between NH and HH. However, HRV analyses need to be conducted carefully as many other factors than autonomic tone affects HRV. These are, for instance, the already mentioned regulation loops as baroreflex or respiratory sinus arrhythmia (Aebi et al., 2020).

### Perspectives

The present study showed once again that normobaric and hypobaric hypoxia are not interchangeable and elicit different responses on physiological parameters (S_p_O_2_) but same HRV responses. Because HRV and hypoxic exposures are increasingly used among athletes (Schmitt et al., 2018), this has important implications. Considering our results, HRV can be used independently of the hypoxic methods for monitoring the athletes’ adaptations to altitude and training loads. However, further investigations, particularly on standing HRV in NH vs. HH, are required before a possible replication of the diagnosis of fatigue sub-category in hypoxia (Schmitt et al., 2015b). Furthermore, standing HRV provides information about the dynamic adaptations of the autonomic nervous system and orthostatic tolerance (Schmitt et al., 2015b). Moreover, since supine and standing HRV are independent (Schmitt et al., 2015a), one cannot be extrapolated from the other, and hypoxia effects on standing HRV remain to be clarified. Further research could also increase the altitude and lengthen the duration of NH and HH exposures. Further research could also increase the altitude and lengthen the duration of NH and HH exposures.

### Strengths and Limitations

On the one hand, this pooling study included more subjects than most other studies in this field, allowing us to reduce the risk of type II errors (false negative). Moreover, the subjects had homogeneous individual characteristics (Table 1). As the eight variables in this study are measured only at three different times, and corrections for multiple comparisons were applied, the risk of type I errors (false positive) remained reasonably low as well. Both hypoxic exposures were performed with rigorous protocol ensuring close experimental conditions and thus limiting external confounding factors. This allowed a targeted and specific analysis on the effects of barometric pressure *per se* in hypoxia. Finally, the perfectly matched control conditions between groups allow an appropriate comparison between two close conditions.

On the other hand, despite that the three protocols performed by our laboratory were quite similar (same crossover design, similar subjects, same measuring equipment and protocol, same data analysis, …), one cannot rule out some potential flaws since the altitude level was different between the three “original” studies. In addition, the present protocols were performed with uncontrolled breathing frequency, known for potentially changing the LF- HF band boundary. However, in our view, this point is not relevant since (1) strictly imposing a breathing frequency may *per se* alter HRV, and (2) this may hide the searched differences in HRV, particularly HF between NH and HH. Moreover, with sensitive variables like HRV, differences in comfort, stress, and environmental conditions such as temperature, humidity, and hypercapnia between exposures may have affected the results (Bernardi et al., 2001).

## Conclusion

Altitude training (alternating “live high – train high” in HH and “live high – train low” in NH) (Millet et al., 2010) and HRV are increasingly used for enhancing and monitoring the athletes’ fatigue and responses to training. In this context, the present study showed that, at least during 24-h hypoxic exposures, the decrease in parasympathetic activity was similar between NH and HH. Therefore, HRV could be analyzed similarly in NH and HH conditions. Furthermore, together with previous results on acute exposures (Aebi et al., 2020), it forms a reliable basis for a similar interpretation of HRV in NH and HH during both acute and long hypoxic exposures.

## Data Availability Statement

The raw data supporting the conclusions of this article will be made available by the authors, without undue reservation.

## Ethics Statement

The studies involving human participants were reviewed and approved by (1) Commission Cantonale Valaisanne d’Ethique Médicale, CCVEM; Agreement 051/09; Sion, Switzerland (2) French National Conference of Research Ethics Committees; N°CPP EST I: 2014/33; Dijon, France. The patients/participants provided their written informed consent to participate in this study.

## Author Contributions

VT wrote the manuscript. RF, JS, and LS conducted the experiments and collected the data. NB was involved in the data analysis. GM conceived and designed the research, revised the manuscript critically, and gave advice to VT for corrections. All authors reviewed and approved the final manuscript before submission.

## Conflict of Interest

The authors declare that the research was conducted in the absence of any commercial or financial relationships that could be construed as a potential conflict of interest.

## Publisher’s Note

All claims expressed in this article are solely those of the authors and do not necessarily represent those of their affiliated organizations, or those of the publisher, the editors and the reviewers. Any product that may be evaluated in this article, or claim that may be made by its manufacturer, is not guaranteed or endorsed by the publisher.

## References

[B1] Aebi M. R. Bourdillon N. Bron D. Millet G. P. (2020). Minimal influence of hypobaria on heart rate variability in hypoxia and normoxia. *Front. Physiol.* 11:1072. 10.3389/fphys.2020.01072 32973566 PMC7472461

[B2] Bärtsch P. Gibbs J. S. R. (2007). Effect of altitude on the heart and the lungs. *Circulation* 116 2191–2202. 10.1161/CIRCULATIONAHA.106.650796 17984389

[B3] Basualto-Alarcón C. Rodas G. Galilea P. A. Riera J. Pagés T. Ricart A. (2012). Cardiorespiratory parameters during submaximal exercise under acute exposure to normobaric and hypobaric hypoxia. *Apunts Med. Esport* 47 65–72. 10.1016/j.apunts.2011.11.005

[B4] Beidleman B. A. Fulco C. S. Staab J. E. Andrew S. P. Muza S. R. (2014). Cycling performance decrement is greater in hypobaric versus normobaric hypoxia. *Extreme Physiol. Med.* 3:8. 10.1186/2046-7648-3-8 24778792 PMC4002198

[B5] Bernardi L. Passino C. Spadacini G. Calciati A. Robergs R. Greene R. (1998). Cardiovascular autonomic modulation and activity of carotid baroreceptors at altitude. *Clin. Sci.* 95 565–573. 10.1042/cs0950565 9791042

[B6] Bernardi L. Sleight P. Bandinelli G. Cencetti S. Fattorini L. Wdowczyc-Szulc J. (2001). Effect of rosary prayer and yoga mantras on autonomic cardiovascular rhythms: comparative study. *BMJ* 323 1446–1449. 11751348 10.1136/bmj.323.7327.1446 PMC61046

[B7] Berntson G. G. Cacioppo J. T. (1999). Heart rate variability: a neuroscientific perspective for further studies. *Card. Electrophysiol. Rev.* 3 279–282. 10.1023/A:1009920002142

[B8] Billman G. E. (2013). The LF/HF ratio does not accurately measure cardiac sympatho-vagal balance. *Front. Physiol.* 4:26. 10.3389/fphys.2013.00026 23431279 PMC3576706

[B9] Bogaard H. J. Hopkins S. R. Yamaya Y. Niizeki K. Ziegler M. G. Wagner P. D. (2002). Role of the autonomic nervous system in the reduced maximal cardiac output at altitude. *J. Appl. Physiol. Bethesda Md.* 1985 271–279. 10.1152/japplphysiol.00323.2001 12070214

[B10] Botek M. Krejèí J. De Smet S. Gába A. McKune A. J. (2015). Heart rate variability and arterial oxygen saturation response during extreme normobaric hypoxia. *Auton. Neurosci. Basic Clin.* 190 40–45. 10.1016/j.autneu.2015.04.001 25907329

[B11] Brooks G. A. Wolfel E. E. Groves B. M. Bender P. R. Butterfield G. E. Cymerman A. (1992). Muscle accounts for glucose disposal but not blood lactate appearance during exercise after acclimatization to 4,300 m. *J. Appl. Physiol.* 72 2435–2445. 10.1152/jappl.1992.72.6.2435 1629100

[B12] Brown T. E. Beightol L. A. Koh J. Eckberg D. L. (1993). Important influence of respiration on human R-R interval power spectra is largely ignored. *J. Appl. Physiol. Bethesda Md* 1985 2310–2317. 10.1152/jappl.1993.75.5.2310 8307890

[B13] Buchheit M. (2014). Monitoring training status with HR measures: do all roads lead to Rome? *Front. Physiol.* 4:73. 10.3389/fphys.2014.00073 24578692 PMC3936188

[B14] Conkin J. (2016). Equivalent air altitude and the alveolar gas equation. *Aerosp. Med. Hum. Perform.* 87 61–64. 10.3357/AMHP.4421.2016 26735235

[B15] Conkin J. Wessel J. H. (2008). Critique of the equivalent air altitude model. *Aviat. Space Environ. Med.* 79 975–982. 10.3357/ASEM.2331.2008 18856188

[B16] Coppel J. Hennis P. Gilbert-Kawai E. Grocott M. P. W. (2015). The physiological effects of hypobaric hypoxia versus normobaric hypoxia: a systematic review of crossover trials. *Extreme Physiol. Med.* 4:2. 10.1186/s13728-014-0021-6 25722851 PMC4342204

[B17] Debevec T. Pialoux V. Poussel M. Willis S. J. Martin A. Osredkar D. (2020). Cardio-respiratory, oxidative stress and acute mountain sickness responses to normobaric and hypobaric hypoxia in prematurely born adults. *Eur. J. Appl. Physiol.* 120 1341–1355. 10.1007/s00421-020-04366-w 32270264

[B18] DiPasquale D. M. Strangman G. E. Harris N. S. Muza S. R. (2015a). Hypoxia, hypobaria, and exercise duration affect acute mountain sickness. *Aerosp. Med. Hum. Perform.* 86 614–619. 10.3357/AMHP.4266.2015 26102141

[B19] DiPasquale D. M. Strangman, Harris N. S. Muza S. R. (2015b). Acute mountain sickness, hypoxia, hypobaria and exercise duration each affect heart rate. *Int. J. Sports Med.* 36 609–614. 10.1055/s-0034-1398623 25837245

[B20] Duplain H. Vollenweider L. Delabays A. Nicod P. Bärtsch P. Scherrer U. (1999). Augmented sympathetic activation during short-term hypoxia and high-altitude exposure in subjects susceptible to high-altitude pulmonary edema. *Circulation* 99 1713–1718. 10.1161/01.CIR.99.13.1713 10190881

[B21] Eckberg D. L. (1983). Human sinus arrhythmia as an index of vagal cardiac outflow. *J. Appl. Physiol.* 54 961–966. 10.1152/jappl.1983.54.4.961 6853303

[B22] Faiss R. Pialoux V. Sartori C. Faes C. Dériaz O. Millet G. P. (2013). Ventilation, oxidative stress, and nitric oxide in hypobaric versus normobaric hypoxia. *Med. Sci. Sports Exerc.* 45 253–260. 10.1249/MSS.0b013e31826d5aa2 22895381

[B23] Fulco C. S. Muza S. R. Beidleman B. A. Demes R. Staab J. E. Jones J. E. (2011). Effect of repeated normobaric hypoxia exposures during sleep on acute mountain sickness, exercise performance, and sleep during exposure to terrestrial altitude. *Am. J. Physiol. - Regul. Integr. Comp. Physiol.* 300 R428–R436. 10.1152/ajpregu.00633.2010 21123763

[B24] Girard O. Brocherie F. Millet G. P. (2017). Effects of altitude/hypoxia on single- and multiple-sprint performance: a comprehensive review. *Sports Med. Auckl. N. Z.* 47 1931–1949. 10.1007/s40279-017-0733-z 28451905

[B25] Goldberg S. Buhbut E. Mimouni F. B. Joseph L. Picard E. (2012). Effect of moderate elevation above sea level on blood oxygen saturation in healthy young adults. *Respiration* 84 207–211. 10.1159/000336554 22441344

[B26] Goldstein D. S. Bentho O. Park M.-Y. Sharabi Y. (2011). Low-frequency power of heart rate variability is not a measure of cardiac sympathetic tone but may be a measure of modulation of cardiac autonomic outflows by baroreflexes: low-frequency power of heart rate variability. *Exp. Physiol.* 96 1255–1261. 10.1113/expphysiol.2010.056259 21890520 PMC3224799

[B27] Grossman P. Taylor E. W. (2007). Toward understanding respiratory sinus arrhythmia: relations to cardiac vagal tone, evolution and biobehavioral functions. *Biol. Psychol.* 74 263–285. 10.1016/j.biopsycho.2005.11.014 17081672

[B28] Hainsworth R. Drinkhill M. J. Rivera-Chira M. (2007). The autonomic nervous system at high altitude. *Clin. Auton. Res.* 17 13–19. 10.1007/s10286-006-0395-7 17264976 PMC1797062

[B29] Hansen J. Sander M. (2003). Sympathetic neural overactivity in healthy humans after prolonged exposure to hypobaric hypoxia. *J. Physiol.* 546 921–929. 10.1113/jphysiol.2002.031765 12563015 PMC2342582

[B30] Hauser A. Schmitt L. Troesch S. Saugy J. J. Cejuela-Anta R. Faiss R. (2016). Similar hemoglobin mass response in hypobaric and normobaric hypoxia in athletes. *Med. Sci. Sports Exerc.* 48 734–741. 10.1249/MSS.0000000000000808 26540262

[B31] Heinzer R. Saugy J. J. Rupp T. Tobback N. Faiss R. Bourdillon N. (2016). Comparison of sleep disorders between real and simulated 3,450-m altitude. *Sleep* 39 1517–1523. 10.5665/sleep.6010 27166242 PMC4945310

[B32] Heistad D. D. Abboud F. M. (1980). Dickinson W. Richards lecture: circulatory adjustments to hypoxia. *Circulation* 61 463–470. 10.1161/01.CIR.61.3.463 7353235

[B33] Hill L. Siebenbrock A. Sollers J. Thayer J. (2009). Are all measures created equal? Heart rate variability and respiration. *Biomed. Sci. Instrum.* 45 71–76. 19369742

[B34] Kember G. C. Fenton G. A. Armour J. A. Kalyaniwalla N. (2001). Competition model for aperiodic stochastic resonance in a Fitzhugh-Nagumo model of cardiac sensory neurons. *Phys. Rev. E* 63:041911. 10.1103/PhysRevE.63.041911 11308881

[B35] Keyl C. Schneider A. Dambacher M. Bernardi L. (2001). Time delay of vagally mediated cardiac baroreflex response varies with autonomic cardiovascular control. *J. Appl. Physiol. Bethesda Md.* 1985 283–289. 10.1152/jappl.2001.91.1.283 11408442

[B36] Kleiger R. E. Stein P. K. Bigger J. T. (2005). Heart rate variability: measurement and clinical utility. *Ann. Noninv. Electrocardiol. Off. J. Int. Soc. Holter Noninvasive Electrocardiol. Inc* 10 88–101. 10.1111/j.1542-474X.2005.10101.x 15649244 PMC6932537

[B37] Koller E. A. Drechsel S. Hess T. Macherel P. Boutellier U. (1988). Effects of atropine and propranolol on the respiratory, circulatory, and ECG responses to high altitude in man. *Eur. J. Appl. Physiol.* 57 163–172. 10.1007/BF00640657 3349981

[B38] Krejèí J. Botek M. McKune A. J. (2018). Dynamics of the heart rate variability and oxygen saturation response to acute normobaric hypoxia within the first 10 min of exposure. *Clin. Physiol. Funct. Imaging* 38 56–62. 10.1111/cpf.12381 27380961

[B39] Liu X. X. Lu L. L. Zhong C. F. Cheng Z. H. Yuan Q. Ren H. R. (2001). [Analysis of heart rate variability during acute exposure to hypoxia]. *Hang Tian Yi Xue Yu Yi Xue Gong Cheng Space Med. Med. Eng.* 14 328–331. 11842848

[B40] Loeppky J. A. Icenogle M. Scotto P. Robergs R. Hinghofer-Szalkay H. Roach R. C. (1997). Ventilation during simulated altitude, normobaric hypoxia and normoxic hypobaria. *Respir. Physiol.* 107 231–239. 10.1016/S0034-5687(97)02523-1 9128904

[B41] Loeppky J. A. Roach R. C. Maes D. Hinghofer-Szalkay H. Roessler A. Gates L. (2005). Role of hypobaria in fluid balance response to hypoxia. *High Alt. Med. Biol.* 6 60–71. 10.1089/ham.2005.6.60 15772501

[B42] Loeppky J. A. Scotto P. Roach R. C. (1996). Acute ventilatory response to simulated altitude, normobaric hypoxia, and hypobaria. *Aviat. Space Environ. Med.* 67 1019–1022. 8908337

[B43] Long M. Qin J. Huang L. Tian K. Yu S. Yu Y. (2006). [Comparison of heart rate variability in healthy young men during exposure to different altitudes]. *Sheng Wu Yi Xue Gong Cheng Xue Za Zhi J. Biomed. Eng. Shengwu Yixue Gongchengxue Zazhi* 23 1195–1197. 17228707

[B44] Malik M. Bigger J. T. Camm A. J. Kleiger R. E. Malliani A. Moss A. J. (1996). Heart rate variability: standards of measurement, physiological interpretation, and clinical use. *Eur. Heart J.* 17 354–381. 10.1093/oxfordjournals.eurheartj.a014868 8737210

[B45] Marshall J. M. (1998). Chemoreceptors and cardiovascular control in acute and chronic systemic hypoxia. *Braz. J. Med. Biol. Res. Rev. Bras. Pesqui. Medicas E Biol.* 31 863–888. 10.1590/s0100-879x1998000700002 9698751

[B46] Maufrais C. Rupp T. Bouzat P. Doucende G. Verges S. Nottin S. (2017). Heart mechanics at high altitude: 6 days on the top of Europe. *Eur. Heart J. Cardiovasc. Imaging* 18 1369–1377. 10.1093/ehjci/jew286 28329216

[B47] Mazzeo R. S. Bender P. R. Brooks G. A. Butterfield G. E. Groves B. M. Sutton J. R. (1991). Arterial catecholamine responses during exercise with acute and chronic high-altitude exposure. *Am. J. Physiol.-Endocrinol. Metab.* 261 E419–E424. 10.1152/ajpendo.1991.261.4.E419 1928333

[B48] Mccraty R. Shaffer F. (2015). Heart rate variability: new perspectives on physiological mechanisms, assessment of self-regulatory capacity, and health risk. *Glob. Adv. Health Med.* 4 46–61. 10.7453/gahmj.2014.073 25694852 PMC4311559

[B49] Millet G. P. Brocherie F. (2020). Hypoxic training is beneficial in elite athletes. *Med. Sci. Sports Exerc.* 52 515–518. 10.1249/MSS.0000000000002142 31939914

[B50] Millet G. P. Debevec T. (2020). CrossTalk proposal: barometric pressure, independent of PO, is the forgotten parameter in altitude physiology and mountain medicine. *J. Physiol.* 598 893–896. 10.1113/JP278673 32053239

[B51] Millet G. P. Faiss R. Pialoux V. (2012). Point: hypobaric hypoxia induces/does not induce different responses from normobaric hypoxia. *J. Appl. Physiol.* 112 1783–1784. 10.1152/japplphysiol.00067.2012 22267386

[B52] Millet G. P. Roels B. Schmitt L. Woorons X. Richalet J. P. (2010). Combining hypoxic methods for peak performance. *Sports Med. Auckl. N. Z.* 40 1–25. 10.2165/11317920-000000000-00000 20020784

[B53] Mounier R. Brugniaux J. V. (2012). Counterpoint: Hypobaric hypoxia does not induce different responses from normobaric hypoxia. *J. Appl. Physiol.* 112 1784–1786. 10.1152/japplphysiol.00067.2012a 22589489

[B54] Naeije R. (2010). Physiological adaptation of the cardiovascular system to high altitude. *Prog. Cardiovasc. Dis.* 52 456–466. 10.1016/j.pcad.2010.03.004 20417339

[B55] Parati G. Ochoa J. E. Torlasco C. Salvi P. Lombardi C. Bilo G. (2015). Aging, high altitude, and blood pressure: a complex relationship. *High Alt. Med. Biol.* 16 97–109. 10.1089/ham.2015.0010 26070056

[B56] Pitzalis M. V. Mastropasqua F. Passantino A. Massari F. Ligurgo L. Forleo C. (1998). Comparison between noninvasive indices of baroreceptor sensitivity and the phenylephrine method in post–myocardial infarction patients. *Circulation* 97 1362–1367. 10.1161/01.CIR.97.14.1362 9577947

[B57] Plews D. J. Laursen P. B. Stanley J. Kilding A. E. Buchheit M. (2013). Training adaptation and heart rate variability in elite endurance athletes: opening the door to effective monitoring. *Sports Med. Auckl. N. Z.* 43 773–781. 10.1007/s40279-013-0071-8 23852425

[B58] Ribon A. Pialoux V. Saugy J. J. Rupp T. Faiss R. Debevec T. (2016). Exposure to hypobaric hypoxia results in higher oxidative stress compared to normobaric hypoxia. *Respir. Physiol. Neurobiol.* 223 23–27. 10.1016/j.resp.2015.12.008 26732282

[B59] Richalet J.-P. (2020). CrossTalk opposing view: barometric pressure, independent of, is not the forgotten parameter in altitude physiology and mountain medicine. *J. Physiol.* 598 897–899. 10.1113/JP279160 32053235

[B60] Richard N. A. Sahota I. S. Widmer N. Ferguson S. Sheel A. W. Koehle M. S. (2014). Acute mountain sickness, chemosensitivity, and cardiorespiratory responses in humans exposed to hypobaric and normobaric hypoxia. *J. Appl. Physiol. Bethesda Md.* 1985 945–952. 10.1152/japplphysiol.00319.2013 23823153

[B61] Ross C. I. Shute R. J. Ruby B. C. Slivka D. R. (2019). Skeletal muscle mRNA response to hypobaric and normobaric hypoxia after normoxic endurance exercise. *High Alt. Med. Biol.* 20 141–149. 10.1089/ham.2018.0147 30994380

[B62] Saugy J. J. Rupp T. Faiss R. Lamon A. Bourdillon N. Millet G. P. (2016a). Cycling time trial is more altered in hypobaric than normobaric hypoxia. *Med. Sci. Sports Exerc.* 48 680–688. 10.1249/MSS.0000000000000810 26559447

[B63] Saugy J. J. Schmitt L. Hauser A. Constantin G. Cejuela R. Faiss R. (2016c). Same performance changes after live high-train low in Normobaric vs. Hypobaric hypoxia. *Front. Physiol.* 7:138. 10.3389/fphys.2016.00138 27148076 PMC4835493

[B64] Saugy J. J. Schmitt L. Fallet S. Faiss R. Vesin J.-M. Bertschi M. (2016b). Sleep disordered breathing during live high-train low in normobaric versus hypobaric hypoxia. *High Alt. Med. Biol.* 17 233–238. 10.1089/ham.2016.0049 27410774

[B65] Saugy J. J. Schmitt L. Cejuela R. Faiss R. Hauser A. Wehrlin J. P. (2014). Comparison of “Live high-train low” in normobaric versus hypobaric hypoxia. *PLoS One* 9:e114418. 10.1371/journal.pone.0114418 25517507 PMC4269399

[B66] Savourey G. Launay J.-C. Besnard Y. Guinet A. Travers S. (2003). Normo- and hypobaric hypoxia: are there any physiological differences? *Eur. J. Appl. Physiol.* 89 122–126. 10.1007/s00421-002-0789-8 12665974

[B67] Schmitt L. Regnard J. Millet G. P. (2015a). Monitoring fatigue status with HRV measures in elite athletes: an avenue beyond RMSSD? *Front. Physiol.* 6:343. 10.3389/fphys.2015.00343 26635629 PMC4652221

[B68] Schmitt L. Regnard J. Parmentier A. Mauny F. Mourot L. Coulmy N. (2015b). Typology of “Fatigue” by heart rate variability analysis in elite nordic-skiers. *Int. J. Sports Med.* 36 999–1007. 10.1055/s-0035-1548885 26252552

[B69] Schmitt L. Regnard J. Coulmy N. Millet G. (2018). Influence of training load and altitude on heart rate variability fatigue patterns in elite nordic skiers. *Int. J. Sports Med.* 39 773–781. 10.1055/a-0577-4429 29902811

[B70] Sevre K. Bendz B. Hankø E. Nakstad A. R. Hauge A. Kåsin J. I. (2001). Reduced autonomic activity during stepwise exposure to high altitude. *Acta Physiol. Scand.* 173 409–417. 10.1046/j.1365-201X.2001.00925.x 11903133

[B71] Shaffer F. McCraty R. Zerr C. L. (2014). A healthy heart is not a metronome: an integrative review of the heart’s anatomy and heart rate variability. *Front. Psychol.* 5:1040. 10.3389/fpsyg.2014.01040 25324790 PMC4179748

[B72] Stray-Gundersen J. Chapman R. F. Levine B. D. (2001). “Living high-training low” altitude training improves sea level performance in male and female elite runners. *J. Appl. Physiol.* 91 1113–1120. 10.1152/jappl.2001.91.3.1113 11509506

[B73] Takezawa T. Dobashi S. Koyama K. (2021). Cardiorespiratory response and power output during submaximal exercise in normobaric versus hypobaric hypoxia: a pilot study using a specific chamber that controls environmental factors. *High Alt. Med. Biol.* 22 201–208. 10.1089/ham.2020.0142 33599547

[B74] Taylor S. E. (2006). Tend and befriend: biobehavioral bases of affiliation under stress. *Curr. Dir. Psychol. Sci.* 15 273–277. 10.1111/j.1467-8721.2006.00451.x

[B75] Wille M. Mairer K. Gatterer H. Philippe M. Faulhaber M. Burtscher M. (2012). Changes in cardiac autonomic activity during a passive 8 hour acute exposure to 5 500 m normobaric hypoxia are not related to the development of acute mountain sickness. *Int. J. Sports Med.* 33 186–191. 10.1055/s-0031-1291325 22290324

[B76] Wolfel E. E. Selland M. A. Mazzeo R. S. Reeves J. T. (1994). Systemic hypertension at 4,300 m is related to sympathoadrenal activity. *J. Appl. Physiol. Bethesda Md.* 1985 1643–1650. 10.1152/jappl.1994.76.4.1643 8045844

